# Does the location of placement of meniscal sutures have a clinical effect in the all-inside repair of meniscocapsular tears?

**DOI:** 10.1186/s13018-017-0591-2

**Published:** 2017-06-09

**Authors:** Uğur Tiftikçi, Sancar Serbest

**Affiliations:** Faculty of Medicine, Department of Orthopaedics and Traumatology, Kırıkkale University, Kırıkkale, Turkey

**Keywords:** Meniscocapsular separation, Arthroscopi, Meniscus repair, All-inside method

## Abstract

**Background:**

Meniscocapsular separation (MCS) is a lesion of the area which is attached from the peripheral section of the meniscus to the capsule and is seen less often than other meniscus injuries. The aim of this study was to investigate which of the different side applications of all-inside MCS repair of the meniscus was better in respect of clinical and functional results.

**Methods:**

In this retrospective study, 53 patients with MCS pattern in their knee joints were treated with arthroscopic meniscus repair made with the all-inside method. The patients were separated into three groups according to the surface from which the fixation was applied: group 1, from the femoral joint surface of the meniscus (*n* = 17), group 2, from the tibial joint surface of the meniscus (*n* = 21) and group 3, from the femoral and tibial joint surfaces of the meniscus (*n* = 15). The participants were assessed using the subjective International Knee Documentation Committee Scoring (IKDC), Lysholm Knee Scale, Tegner Activity Level Scale, Barrett criteria and Kellgren–Lawrence classification after a 45 ± 12.1 months (range, 24–70 months) follow-up.

**Results:**

Postoperatively, all the groups exhibited significantly increased subjective IKDC score, Lysholm score and Tegner activity score compared with their preoperative results (*p* < 0.001). At 6 months postoperatively, a statistically significant difference was determined between the groups in respect of the subjective IKDC score, Tegner activity score and Lysholm score with group 2 showing better results than the other groups (*p* < 0.001). At the final follow-up examination, no statistically significant difference was determined between the groups in respect of the subjective IKDC score, Tegner activity score or Lysholm score. A statistically significantly lower level of pulling and stress sensation was determined in group 2 (*p* < 0.001).

**Conclusions:**

MCS repair made with the all-inside method is successful clinically and functionally and in respect of MRI findings. In addition, it was seen that the fixation method applied from the tibial surface of the meniscus does not disturb the anatomic position of the meniscus in MCS repair. The tibial joint surface is the most appropriate area for suturation in all-inside repair of MCS.

**Level of evidence:**

Level IV.

## Background

Meniscocapsular separation (MCS) is a lesion of the area which is attached from the peripheral section of the meniscus to the capsule and is seen less often than other meniscus injuries [22, 25]. MCS is most often in the posterior horn section, is often a lesion in the meniscotibial ligament and accompanies microtrauma and anterior cruciate ligament (ACL) tears [1, 4, 13]. Several studies have reported a high rate of recovery after MCS repair [9, 10, 12]. As the blood circulation is good in the meniscocapsular region and in the peripheral third of the meniscus, studies have reported that the recovery rate is high and the re-operation risk is low [18, 19]. MCS is repaired with inside-out and all-inside methods. Each method has its own advantages and disadvantages. MCS inside-out repair is the gold standard in respect of biomechanical strength. However, the disadvantage is that posteromedial and posterolateral cuts are required [5, 16, 17, 20, 21]. Although inside-out techniques are the gold standard in MCS repair, current applications are all-inside suturation techniques. The most significant advantages of MCS repair with new all-inside devices are that it can be easily and quickly applied and that there is no need for an extra cut [8, 12]. Disadvantages are neurovascular injuries and that the anterior meniscus is not suitable for suturation. Another problem is caused by the elevation of the meniscus to the femoral side [27]. In these patients, there is a greater pulling and stress sensation, especially when the knee moves from the flexion to extension. This symptom can be caused by exposure to greater stress because of the change in the anatomic orientation in the function of dynamic stability of the meniscocapsular complex.

The primary aim of this study was to report the clinical and functional results of MCS all-inside repair. The secondary aim was to investigate which of the different side applications of all-inside repair of the meniscus was better in respect of the clinical and functional results.

## Methods

**Fig. 1:**
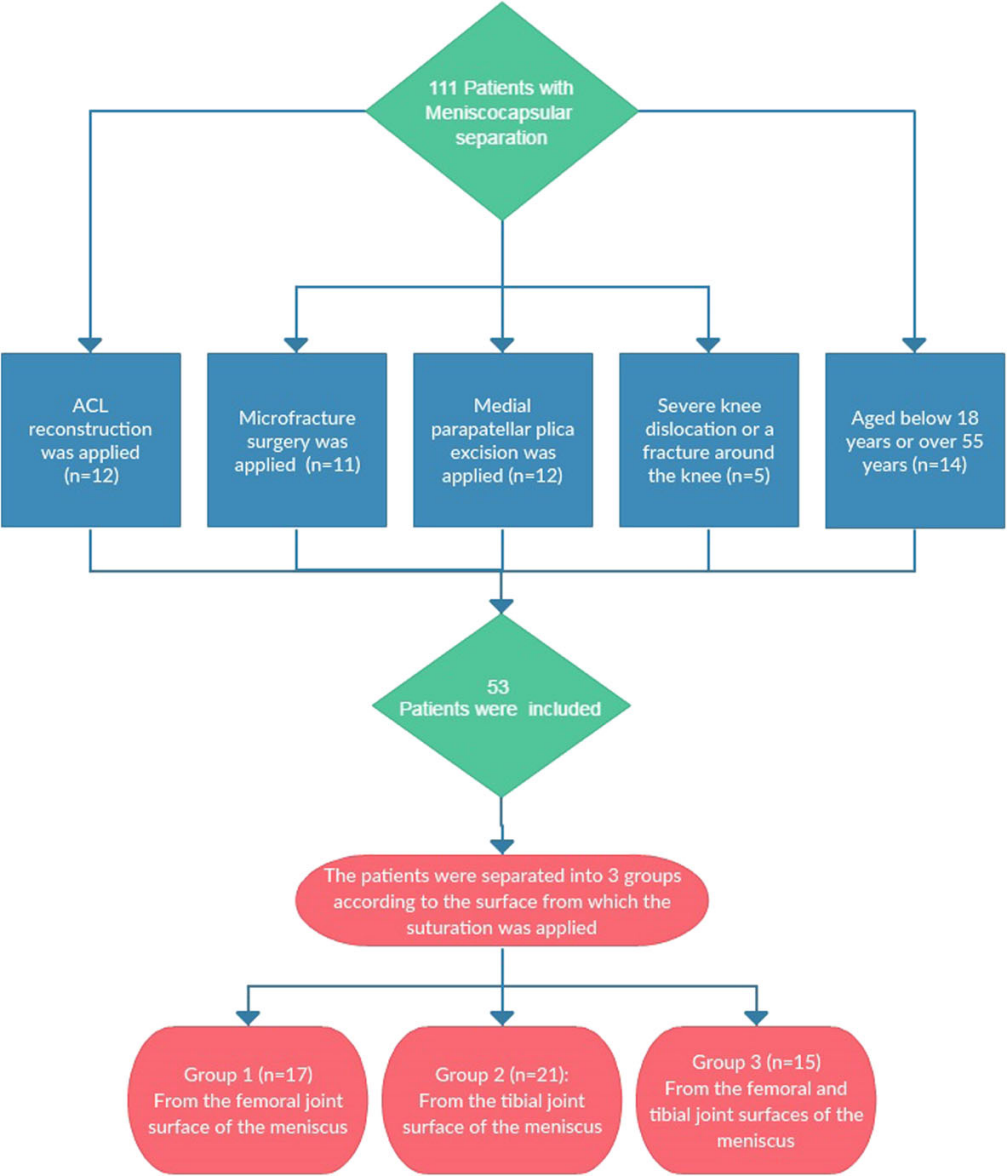
Flow diagram of exclusiın criteria

The study included 111 patients with MCS pattern who underwent meniscus repair in the Orthopaedics and Traumatology Clinic between 2009 and 2014. Approval for the study was granted by the Local Ethics Committee (2014/23). A retrospective review was made of the patient files. Criteria for inclusion in the study were that patients were determined with MCS arthroscopically or with findings of MCS on magnetic resonance imaging (MRI) which were in the zones 3 and 4 posterior of the meniscus, had not benefitted from more than 3 months of physical therapy, were aged over 18 years and below 55 years and were followed up for at least 24 months. Patients excluded from the study for the reasons mentioned above were as follows: ACL reconstruction was applied (20 cases), microfracture surgery was applied (11 case), medial parapatellar plica excision was applied (12 cases), severe knee dislocation or a fracture around the knee (5 cases), aged below 18 years or over 55 years (fourteen cases), follow-up of less than 24 months (16 cases). Fifty three patients met the inclusion criteria and were therefore included in the study (Fig. 1).

The patients were separated into three groups according to the surface from which the suturation was applied: group 1 (*n* = 17), from the femoral joint surface of the meniscus, group 2 (*n* = 21), from the tibial joint surface of the meniscus and group 3 (*n* = 15), from the femoral and tibial joint surfaces of the meniscus (Fig. 1).

A record was made for all patients of symptoms (locking, pain, swelling, postoperative pulling sensation), age, gender, whether pain was acute (<3 months) or chronic (>3 months), MRI diagnosis, location of the meniscus tear, MCS length, number of sutures used, comorbidities, postoperative complications or side-effects, requirement for postoperative drainage, preoperative and postoperative (6 months and final 24 months follow-up examination) subjective International Knee Documentation Committee Scoring (IKDC), Lysholm Knee Scale [14], Tegner Activity Level Scale [26], Barrett criteria (joint area, effusion, McMurray test sensitivity) [2] and Kellgren–Lawrence classification for degenerative arthritis [11]. All the preoperative and postoperative MR images (Achieva 1.5T MRI system-Philips Medical Systems, Best, The Netherlands) were evaluated by a radiologist experienced in the musculoskeletal system.

### Surgical technique

**Fig. 2:**
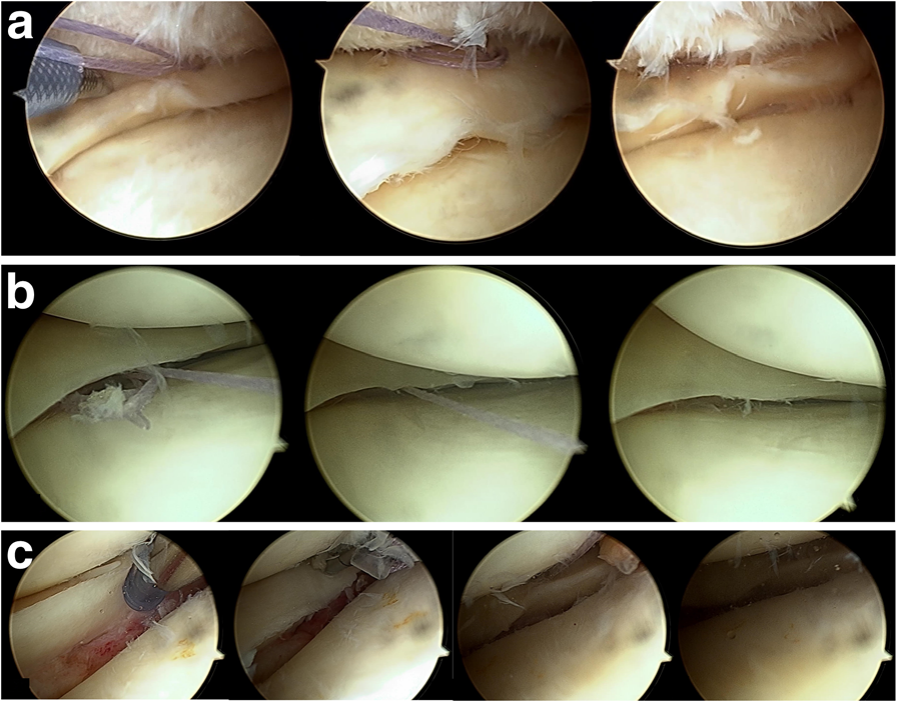
The all-inside meniscal repair was made according to the options of application from the femoral joint surface of the meniscus (**a**), from the tibial joint surface of the meniscus (**b**) and from the femoral and tibial joint surfaces of the meniscus (**c**)

With the patient in the standard knee arthroscopy position, entry to the knee was made from the anterolateral and anteromedial portals. Meniscus tear was determined arthroscopically. The tear ends were cleaned in the meniscocapsular region with a shaver and rasp. All-inside meniscus repair was applied to all the patients with MCS lesion. The all-inside meniscal repair was made according to the options of application from the femoral, tibial and femoral-tibial surfaces of the meniscus (Fig. 2). The Omnispan Meniscal Repair System (Mitek, Norwood, MA, USA) all-inside suture device was used for meniscal repair. In arthroscopy, the size and location of the meniscus tear, the number of sutures used and the status of other anatomic structures (ACL injuries, cartilage injuries, parapatellar plica) in the knee were recorded.

### Postoperative rehabilitation

All patients started early postoperative patellar mobilisation exercises with isometric quadriceps exercises, and walking was permitted with two crutches non-weight-bearing. Passive knee joint exercises up to 90° were applied for 2 weeks postoperatively, and after 2 weeks, full joint movement was permitted. After the fourth week, unassisted walking with weight-bearing as tolerated was permitted and full joint movement exercises were started. In posterior meniscus tears, active knee flexion over 90° and squatting and sitting on the floor were not permitted for up to 6 weeks. At 3 months after arthroscopy, patients with no knee complaints (swelling, pulling, locking, pain and negative McMurray test) were permitted to recommence active sports and high-level activities.

### Statistical analysis

All statistical analyses were performed with SPSS ver. 16.0 (SPSS Inc., Chicago, IL, USA). A confidence interval (CI) of 95% and a two-tailed *p* < 0.05 were determined to be statistically significant for all of the analyses. The distance between the center of the talus and each landmark was expressed as mean and SD. One-way ANOVA with Turkey–Kramer test was used for the comparison among landmarks. Statistical significant was set at *p* < 0.05.

## Results

The mean age of the total 53 patients was 37.6 ± 9.4 years (range, 20–55 years). Repair was made to the right knee of 28 patients, the left knee of 25 patients, in the medial meniscus in 47 cases, the lateral meniscus in 6 and to traumatic tears in 37 cases. The demographic characteristics and preoperative examination findings of the groups are shown in Table 1. The mean follow-up period was 45 ± 12.1 months (range, 24–70 months). Preoperatively, there were no findings of MCS in 14 cases. No major complication developed in any patient intra-operatively or in the early postoperative period. No statistically significant difference was determined functionally between the groups in respect of the preoperative subjective IKDC score, Lysholm Knee Scale score and Tegner Activity Level Scale score. At 6 months postoperatively, a statistically significant difference was determined between the groups in respect of the subjective IKDC score, Lysholm Knee Scale score and Tegner Activity Level Scale score with group 2 (tibial side) showing better results than the other groups (*p* < 0.001). At the final 24 months follow-up examination, no statistically significant difference was determined between the groups in respect of the subjective IKDC score, Lysholm Knee Scale score and Tegner Activity Level Scale score (Table 2).

**Table 1:** The demographic characteristics and preoperative examination findings of the groups

	Total (*n* = 53)	Group 1 (*n* = 17)	Grup 2 (*n* = 21)	Grup 3 (*n* = 15)
Age (mean ± SD)	37.6 ± 9.4	36.5 ± 10.1	36.5 ± 9.0	40.4 ± 9.4
Gender M/F	36/17	13/4	13/8	10/5
Extremity side–right/left	28/25	7/10	13/8	8/7
Meniscus side M/L	47/6	16/1	18/3	13/2
Follow-up (mean ± SD)	45.0 ± 12.1	50.7 ± 11.8	42.3 ± 10.6	42.3 ± 12.3
MRI lesion present/absent	39/14	12/5	18/3	9/6
McMurray test	42	14	17	11
Effusion	27	9	11	7
Joint sensitivity	38	12	13	13

**Table 2:** Functional results

Evaluation method	Preoperative scores	Postoperative scores 6 months	Postoperative scores 24 months	Postoperative 6 months				Postoperative 24 months			
				*p*	*p* (1-2)	*p* (1-3)	p (2-3)	p	p (1-2)	p (1-3)	p (2-3)
Lysholm	61.19 ± 12.64	79.75 ± 7.56	90.92 ± 4.54	*<0.001*	*<0.001*	>0.05	*<0.001*	*<0.001*	>0.05	>0.05	>0.05
Group 1	59.24 ± 11.37	77.12 ± 6.56	89.94 ± 5.64								
Group 2	61.71 ± 12.75	84.95 ± 4.89	91.76 ± 4.02								
Group 3	62.67 ± 14.37	75.47 ± 7.76	90.87 ± 3.88								
Tegner	3.43 ± 1.43	4.92 ± 1.28	6.23 ± 1.52	*<0.001*	*<0.001*	>0.05	*<0.001*	*<0.001*	>0.05	>0.05	>0.05
Group 1	3.18 ± 1.38	4.76 ± 1.20	6.59 ± 1.80								
Group 2	3.86 ± 1.42	5.43 ± 1.28	6.19 ± 1.43								
Group 3	3.13 ± 1.45	4.40 ± 1.18	5.87 ± 1.30								
IKDC	50.45 ± 10.06	78.98 ± 5.78	91.93 ± 4.90	*<0.001*	*<0.001*	>0.05	*<0.001*	*<0.001*	>0.05	>0.05	>0.05
Group 1	50.35 ± 8.27	75.18 ± 6.64	88.09 ± 4.33								
Group 2	50.68 ± 5.20	82.18 ± 3.30	95.65 ± 2.80								
Group 3	55.77 ± 8.33	78.82 ± 5.00	91.06 ± 4.14								

A pulling and stress sensation was present in 12 of the 17 patients in group 1 and continued for up to 6 months, in 3 of the 21 patients in group 2 and in 7 of the 15 patients in group 3. A statistically significantly lower level of pulling and stress sensation was determined in group 2 (*p* < 0.001).

Degeneration was classified according to the Kellgren–Lawrence grading system on the final radiographs. These were determined as grade 0 in 42, grade 1 in 8, grade 2 in 2 and grade 3 in the 0 knees (Table 3). No statistically significant difference was determined between the groups (*p* > 0.05). Insufficient recovery was determined postopertively in two patients. Clinically, there was no effusion in these two patients. In one, there was sensitivity in the joint line, and in the other, the Mc Murray test [15] was positive. As the symptoms were mild, re-operation was not considered for either patient.

**Table 3:** Preoperative and postoperative Kellgren–Lawrence OA classification scores

	Total (*n* = 53)		Group 1 (*n* = 17)		Grup 2 (*n* = 21)		Grup 3 (*n* = 15)	
	Preoperative scores	Postoperative scores 24 months	Preoperative scores	Postoperative scores 24 months	Preoperative scores	Postoperative scores 24 months	Preoperative scores	Postoperative scores 24 months
Kellgren–Lawrence grading								
Grade 0/1/2/3	47/6/0/0	42/8/2/1	15/2/0/0	12/3/1/1	19/2/0/0	17/3/1/0	13/2/0/0	13/2/0/0

In all the patients, a significant improvement was determined in the functional scores compared to the preoperative values. The feeling of pulling and stress was determined to be lower in group 2, where MCS repair was made from the tibial section of the meniscus. The functional scores of the knee in the early postoperative period were also seen to be better in group 2. No statistically significant difference was determined between the groups in respect of the other variables evaluated of age, gender, side, number of sutures, traumatic or chronic, size of the tear, ACL status or comorbidities.

## Discussion

The most important point of MCS is that care must be taken during MRI and arthroscopy and the diagnosis should not be overlooked. In this study, the mid-term clinical results were extremely good and the anatomic and early clinical results of the application of all-inside suturing from the tibial side were better than those of the other groups.

MCS is a lesion separating the meniscus from the meniscus joint capsule, and as the severity of the trauma increases, a longitudinal tear pattern occurs. It generally accompanies microtrauma and ACL tears [1, 4, 13]. In MCS, the lesion is often in the meniscotibial ligament. The meniscotibial ligament attaches the medial meniscus to the tibia and plays an important role in static and dynamic stability of the knee. In a cadaver study by Dugas et al. [6] related to the contact of MCS lesion with biomechanics, no significant difference was found in the transfer of pressure. It was also determined that there was a tendency to return to normal after MCS repair; although, the knee was not tested at different flexion angles. That study demonstrated that the meniscocapsular complex played an important role more in dynamic stability. When the meniscotibial ligament is involved in the lesion, it cannot undertake the function of braking stabilisation on the femoral condyle and thus causes instability [3, 7, 24]. This is also one of the most significant causes of MCS lesion. Therefore, MCS lesion repair plays an important role in knee stability. In parallel with this knowledge, it is possible to place the sutures from the tibial side in the all-inside method of meniscotibial ligament repair in MCS lesion. In a repair made from the tibial side of the meniscus, the anatomical position of the meniscus is better [27].

There are very few studies in literature reporting repair with all-inside suture materials because of MCS [9, 10, 12]. Hirtler et al. reported that all-inside repair was a safe and advantageous treatment option in the repair of acute and chronic MCS in young athletes [10]. Li et al. reported satisfactory results in patients applied with ACL reconstruction and all-inside arthroscopic repair of MCS tear of the posterior horn of the meniscus [12]. In the current study, with the exception of two patients, good recovery was determined in all the other patients at the final follow-up examination according to the clinical scores, functional scores (IKDC, Lysholm score) and MRI. In the patients of the current study where repair was applied from the femoral surface of the meniscus, the postoperative congruence was less satisfactory and the knee functions were seen to improve later.

If the MCS lesion is <5 mm, it may not be able to be diagnosed on MRI and diagnosis can be made arthroscopically in these patients. The history, physical examination, chronic medial knee pain and careful arthroscopic viewing are very important in these patients [9]. In a study by Sonnery et al., it was reported that occult lesions of the medial meniscus are extremely frequent and diagnosis can be made more easily with debridement of the synovial tissues in the meniscocapsular region [23]. In the current study, there were no findings of MCS on preoperative MRI in 14 (26%) patients. In 11 of these patients, there was ACL lesion and meniscocapsular separation was diagnosed during arthroscopy and then repaired. In the other eight patients, diagnosis was able to be made during arthroscopy applied because of persistent joint pain, degenerative joint and other lesions in the meniscus. In arthroscopy applied because of ACL lesion, the presence of MCS in particular must be investigated.

As reported in a study by Tiftikci and Serbest, in a repair made with an all-inside suture device, femoral elevation of the meniscus impairs the normal anatomic placement [27]. In these patients, there is a greater pulling and stress sensation in the area where suturation is applied. To the best of our knowledge, there are no studies in literature related to this pulling and stress sensation. In the postoperative follow-up of patients applied with all-inside repair of MCS, pulling and stress sensation is a significant clinical problem which causes discomfort to patients. In the current study, the pulling and stress sensation was observed more often in the group where repair was made from the femoral joint surface of the meniscus. The group where these symptoms were observed the least was the group where repair was made from the tibial surface of the meniscus.

The most significant limitation of this study is that it was retrospective. Secondly, the number of patients was low. Thirdly, there was no comparison of the all-inside repair method with the inside-out or outside-in methods. Finally, the pulling and stress sensation in the knee could have been caused by incorrect placement related to the medial-lateral collateral ligament of the all-inside peak diameters.

## Conclusions

The results of this study showed that MCS repair made with the all-inside method is successful clinically and functionally and in respect of MRI findings. In addition, it was seen that the suturation method applied from the tibial surface of the meniscus does not disturb the anatomic position of the meniscus in MCS repair. Furthermore, the optimum conditions are provided for the restoration of the functions of the meniscus and there is a lower rate of symptoms such as pulling and stress in the postoperative period. The tibial joint surface is the most appropriate area for suturation in all-inside repair of MCS.

## Abbreviations

ACL: Anterior cruciate ligament
IKDC: International Knee Documentation Committee
MCS: Meniscocapsular separation
MRI: Magnetic resonance imaging
